# An Autonomous Star Identification Algorithm Based on One-Dimensional Vector Pattern for Star Sensors

**DOI:** 10.3390/s150716412

**Published:** 2015-07-07

**Authors:** Liyan Luo, Luping Xu, Hua Zhang

**Affiliations:** School of Aerospace Science and Technology, Xidian University, Xi’an 710126, China; E-Mail: liyanluo@stu.xidian.edu.cn

**Keywords:** star sensor, star identification, one-dimensional vector pattern, star pattern

## Abstract

In order to enhance the robustness and accelerate the recognition speed of star identification, an autonomous star identification algorithm for star sensors is proposed based on the one-dimensional vector pattern (one_DVP). In the proposed algorithm, the space geometry information of the observed stars is used to form the one-dimensional vector pattern of the observed star. The one-dimensional vector pattern of the same observed star remains unchanged when the stellar image rotates, so the problem of star identification is simplified as the comparison of the two feature vectors. The one-dimensional vector pattern is adopted to build the feature vector of the star pattern, which makes it possible to identify the observed stars robustly. The characteristics of the feature vector and the proposed search strategy for the matching pattern make it possible to achieve the recognition result as quickly as possible. The simulation results demonstrate that the proposed algorithm can effectively accelerate the star identification. Moreover, the recognition accuracy and robustness by the proposed algorithm are better than those by the pyramid algorithm, the modified grid algorithm, and the LPT algorithm. The theoretical analysis and experimental results show that the proposed algorithm outperforms the other three star identification algorithms.

## 1. Introduction

Celestial navigation has a broad application prospect in the automated sky survey system and the deep space exploration. Star sensors have good autonomy, high precision and work reliably, and they play an important role in the celestial navigation. In the “lost-in-space” mode, star sensors can automatically determine the spacecraft attitude [1,2,3] without *a priori* attitude information. The performance of the star identification algorithm directly affects the attitude determination of the spacecraft. The recognition accuracy of the star identification algorithm affects the system precision of star sensor, and the recognition speed of star identification affects the responsiveness of star sensor. Therefore, a rapid and robust star identification algorithm is necessary.

During the past few decades, many star identification algorithms have been created which are widely used in the spacecraft attitude determination and control when no *a priori* attitude information is available [4]. The star identification algorithms mainly include the polygon algorithm [5,6,7,8,9,10,11,12,13,14,15], the match group algorithm [16], the grid algorithm [17,18,19,20,21], the neural network algorithm [22,23,24], the genetic algorithm [25,26], and so on, which can be roughly classified into two basic categories [27]: subgraph isomorphism and pattern recognition.

The triangle algorithm [10,11] is a classical star identification algorithm and many other star identification algorithms are derived from it. In the triangle algorithm, every three observed stars forms a triangle and its angular distances are adopted as the feature for star identification. However, there are many stars existing at small angular distances which are difficult to distinguish from each other under the noise interference. In addition, the angular distance is sensitive to noise, which is easy to cause mismatching. The polygon algorithm is derived from the triangle algorithm. Since the dimension of the identification feature is limited in the polygon algorithm, a lot of the information of the stars observed in the field of view (FOV) cannot be efficiently used. Although the increase of the dimension of the identification feature can improve the utilization of the stars’ information, the recognition algorithm is complicated which is not good for star identification. Mortari *et al.* [7] used five observed stars to increase the dimension of the identification feature, which can reduce the redundant matching to some extent. Moreover, some improved algorithms have been proposed to solve the existing problem in the triangle star identification algorithm [12,13,14,15]. But the inherent shortcomings of the triangle algorithm cannot be solved thoroughly.

The pattern-based star identification algorithm has incomparable advantages compared with the subgraph-isomorphism-based star identification algorithm, and much effort has been made in research about the pattern-based star identification.

In 1997, Padgett *et al.* [17] proposed the grid algorithm for autonomous star identification in which the star recognition problem is converted into the comparison of the two 0–1 strings. In the grid algorithm, multiple pixels are divided into a grid cell, so this algorithm is insensitive to the positional noise. But the measurement precision of the magnitude and the choice of the subaltern star have great impact on the accuracy and reliability of the grid algorithm. Lee *et al.* [18,19,20,21] proposed a modified grid algorithm in which the recognition accuracy is better than that of the original one. Both the original grid algorithm and the modified grid algorithms need to store the entire star pattern, so they require more memory space to store the feature database. In addition, the size of the feature database increases with the increase of the grid cells.

Wei *et al.* [28] used the Log-Polar transform to convert the rectangular coordinate system into the polar coordinate system in which the translation and rotation of the observed stars is simplified as the displacement operation. The LPT algorithm has a strong anti-interference ability against position noise and the magnitude noise. But a lot of time will be taken in the search for the matching star pattern of the observed star pattern in the feature database. In recent years, some novel star identification algorithms have been proposed, such as the singular value method [29], the vector pattern matching method [30], the pattern code method [31], and so on [16,32,33,34,35,36,37,38]. These algorithms have the same goal which is to identify the stars as quickly and robustly as possible and they solve the problem of star identification in different aspects. However, there still is no optimal star identification algorithm to date.

In order to reduce the time-consumed and enhance the robustness of star identification, we propose an autonomous star identification algorithm based on the one-dimensional vector pattern. In this method, the main star and the alignment star are chosen to reset the new coordinate axes firstly. That makes it possible to establish the one-to-one relationship between every navigation star and its one-dimensional vector pattern. Secondly, all stars observed in FOV are projected onto the new coordinate axes using the rotation matrix. Then the observed stars, locating in the neighboring region of the main star, are used to build the one-dimensional vector pattern of the main star. Finally, the feature vector of the main star can be achieved according to the one-dimensional vector pattern.

The one-dimensional vector pattern not only describes the position information of the observed stars, but also expresses the angular information between the observed stars and the horizontal axis. The one-dimensional vector pattern can fully express the space geometry information of the stars observed in FOV, which has greatly contributed to the star identification. In addition, the one-dimensional vector pattern of the same navigation star remains unchanged when the stellar image rotates, so there is a one-to-one relationship between the navigation star and its one-dimensional vector pattern, which makes it possible to identify the observed star quickly.

The rest of this paper is organized as follows. In Section 2, the one-dimensional vector pattern is introduced in detail. The star pattern generation and the identification process are described in Section 3. The simulation conditions are introduced in Section 4. In Section 5, the simulation results and the numerical analysis are given. Finally, in Section 6, the concluding remarks are made.

## 2. Description of the One-Dimensional Vector Pattern

Generally, the position information and the magnitude of the navigation star are considered as the basic characteristics of the navigation star. In many star identification algorithms, the position information is used to build the geometric feature to identify the navigation star. The magnitude of the navigation star also can be used to identify the navigation star. However, the magnitude is unstable and it is used only for the rough estimation. In this paper, only the position information of the navigation star is used to identify the navigation star.

### 2.1. The Imaging Principle in Star Sensor

**Figure 1:**
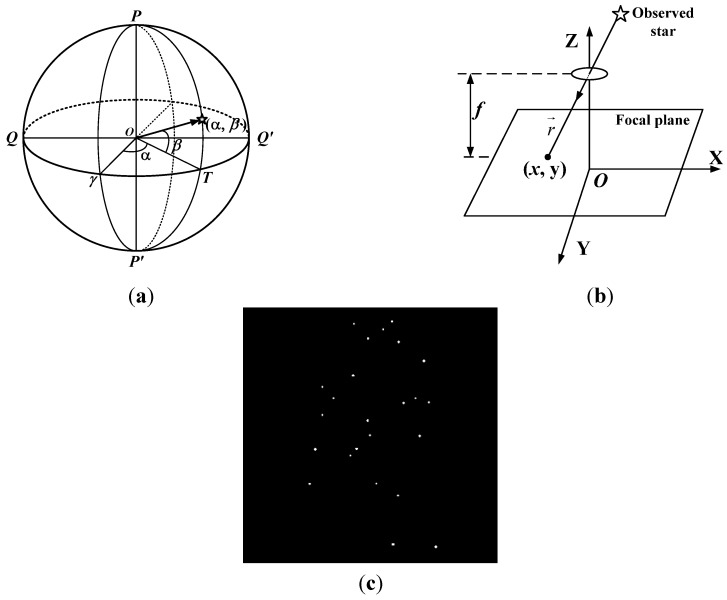
Stellar image in star sensor. (**a**) Celestial sphere reference frame; (**b**) The imaging principle in star sensor; (**c**) The stars observed in FOV.

The star catalog and astronomical almanac express a star’s position in terms of its right ascension α and declination β in the celestial sphere reference frame (see Figure 1a). The parallel light from stars is imaging on the focal plane of the charge-coupled device (CCD) in star sensor, so the position of the star in the celestial sphere reference frame is expressed in terms of pixels along the *x* and *y* axes on the stellar image (see Figure 1b). The light points on the stellar image are the stars observed in FOV (see Figure 1c), which are the research objects in star identification. Before the experiment, it needs to simulate the stellar image according to the information of the navigation stars in the star catalogue.

Generally, only the plane position of the observed star can be achieved in star sensor. During the process of star recognition, the geometric position relationships among the observed stars are adopted to identify the positions of the observed stars in the celestial sphere reference frame. In the simulation experiments, the right ascension and declination of the navigation star in the celestial sphere reference frame need to be converted into the plane coordinates firstly. The transformation relation can be expressed as

(1) $$\begin{array}{l} x_{r}=\frac{N_{x}\;\text{cos}\;\beta \;\text{sin}(\alpha_{i}\;-\;\alpha )}{2\;\text{tan}(FOV_{x}\;/\;2)(\text{sin}\;\beta_{i}\;\text{sin}\;\beta \;+\;\text{cos}\;\beta_{i}\;\text{cos}\;\beta \;\text{cos}(\alpha_{i}\;-\;\alpha ))}\\ y_{r}=\frac{N_{y}\;(\text{sin}\;\beta_{i}\;\text{cos}\;\beta -\text{cos}\;\beta_{i}\;\text{sin}\;\beta \;\text{cos}(\alpha_{i}-\alpha ))}{2\;\text{tan}(FOV_{y}\;/\;2)(\text{sin}\;\beta_{i}\;\text{sin}\;\beta \;+\;\text{cos}\;\beta_{i}\;\text{cos}\;\beta \;\text{cos}(\alpha_{i}\;-\;\alpha ))} \end{array}$$

where (*N_x_*, *N_y_*) is the resolution of CCD in star sensor, (*FOV_x_*, *FOV_y_*) is the FOV of CCD, (α_*i*_, β_*i*_) is the right ascension and declination of the *i*th observed star, and (α, β) is the optical axis direction of CCD. The optical axis direction of CDD points to the position of the star in the celestial sphere reference frame, which is projected on the center of the stellar image. The centroid coordinates (*x_r_*, *y_r_*) are used as the actual centroid coordinates of the navigation star in the simulation experiments.

### 2.2. The One-Dimensional Vector Pattern

**Figure 2:**
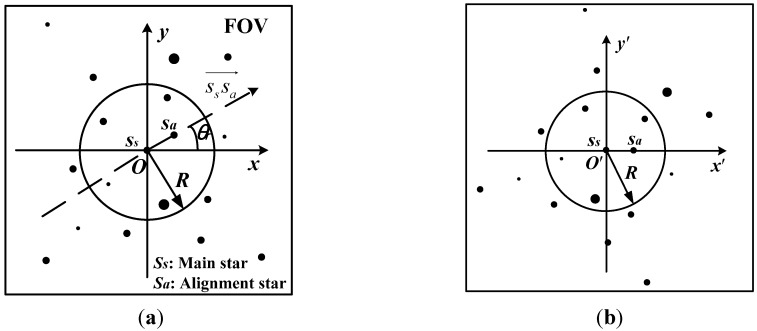
The vector direction of the star pattern. (**a**) The alignment star; (**b**) The stellar image is rotated.

Aiming at the improvement of the speed of star identification, the one-to-one relationship between the navigation star and its one-dimensional vector pattern is built in the proposed algorithm. In order to achieve the unique pattern of the navigation star, the centroid coordinates of stars observed in FOV are reset. In the process of star identification, one of the observed stars is chosen as the main star, and the observed stars located in the neighboring region with a radius of *R* (see Figure 2) are called the neighbor stars of the main star. The star pattern of the main star consists of the main star and its neighbor stars. The nearest neighbor star is regarded as the alignment star of the main star. The direction from the main star to the alignment star is considered as the vector direction of the star pattern.

In order to achieve the unique star pattern when the stellar image rotates, the centroid coordinates of the observed stars are reset based on the vector direction of the star pattern. The centroid coordinates of the star observed in FOV is expressed as (*x*, *y*) on the stellar image in *oxy* coordinates. The centroid coordinates of the main star and the alignment star are set to be (*x_s_*, *y_s_*) and (*x_a_*, *y_a_*), respectively. The direction from the main star to the alignment star is expressed as $\vec{s_{s}s_{a}}$, which is denoted as the *o*ʹ*x*ʹ axis among the new coordinate axes (see Figure 2). The plane included angle θ_*r*_ between the *ox* axis and the *o*ʹ*x*ʹ axis is expressed as

(2) $$\theta_{r}=\langle \vec{s_{s}s_{a}},\vec{ox}\rangle =\text{arctan}(\frac{y_{a}-y_{s}}{x_{a}-x_{s}})$$

The plane included angle θ_*r*_ is set to be positive from the *ox* axis to $\vec{s_{s}s_{a}}$ on counterclockwise. The stellar image is rotated with the angle of θ_*r*_, and the vector direction is denoted as the *o*ʹ*x*ʹ axis. The right-hand rule is used to obtain the *o*ʹ*y*ʹ axis, and then the centroid coordinates of the stars are expressed in terms of pixels along *x*ʹ and *y*ʹ axes in the *o*ʹ*x*ʹ*y*ʹ coordinates. The new centroid coordinates of the observed stars in the *o*ʹ*x*ʹ*y*ʹ coordinates will be achieved using Equation (3).

(3) $$\left[\begin{array}{l} x^{\prime }\\ y^{\prime } \end{array}\right]=\left[\begin{array}{l} \text{cos}\theta_{r}\;\;\;\;\;\;\;\;\;\;\;\text{sin}\theta_{r}\\ -\text{sin}\theta_{r}\;\;\;\;\;\;\;\;\;\text{cos}\theta_{r} \end{array}\right]\left[\begin{array}{l} x\\ y \end{array}\right]+\left[\begin{array}{l} \Delta x\\ \Delta y \end{array}\right]\;=M\left[\begin{array}{l} x\\ y \end{array}\right]+\left[\begin{array}{l} \Delta x\\ \Delta y \end{array}\right]$$

where (*x*, *y*) are the centroid coordinates of the observed star in the *oxy* coordinates, and (*x*ʹ, *y*ʹ) are the new centroid coordinates of the corresponding observed star in the *o*ʹ*x*ʹ*y*ʹ coordinates, and *M* is the rotation matrix. The offsets between the origin of the *oxy* coordinates and the origin of the *o*ʹ*x*ʹ*y*ʹ coordinates can be expressed as

(4) $$\Delta x=x_{0}-x_{s},\Delta y=y_{0}-y_{s}$$

where (*x*_0_, *y*_0_) is the origin of the *oxy* coordinates. Generally, *x*_0_ = 0 and *y*_0_ = 0, so the offsets can be rewrote as

$$\Delta x=-x_{s},\Delta y=-y_{s}$$

As described above, the new centroid coordinates of the observed stars can be achieved using Equation (5), when the stellar image rotates with the plane included angle of θ_*r*_.

(5) $$\left[\begin{array}{l} x^{\prime }\\ y^{\prime } \end{array}\right]=\left[\begin{array}{l} \text{cos}\theta_{r}\;\;\;\;\;\;\;\;\;\;\;\text{sin}\theta_{r}\\ -\text{sin}\theta_{r}\;\;\;\;\;\;\;\;\;\text{cos}\theta_{r} \end{array}\right]\left[\begin{array}{l} x\\ y \end{array}\right]+\left[\begin{array}{l} -x_{s}\\ -y_{s} \end{array}\right]\;=M\left[\begin{array}{l} x\\ y \end{array}\right]+\left[\begin{array}{l} -x_{s}\\ -y_{s} \end{array}\right]$$

The relative position of every two observed stars remains unchanged when the stellar image rotates. The main star locates on the origin of the *o*ʹ*x*ʹ*y*ʹ coordinates. The horizontal axis coordinates of the observed stars in the star pattern indicate the positions of these stars on the *o*ʹ*x*ʹ axis (see Figure 2a). Set the plane included angle between the neighbor star and the alignment star as θ, and the plane included angle along the counterclockwise as positive (see Figure 2b). The positions and the plane included angles of the observed stars in the star pattern are adopted as the characteristics of the star pattern. The one-dimensional vector pattern of the star pattern can be expressed

(6) $$V=\left\{\left(x_{1}^{\prime },\theta_{1}\right),\ldots ,\left(x_{n}^{\prime },\theta_{n}\right)\right\}=\left\{\left(x_{i}^{\prime },\theta_{i}\right)\right\},i=1,\ldots ,n$$

where $x_{i}^{\prime }$ is the horizontal axis coordinate of the *i*th neighbor star, and θ_*i*_ is the plane included angle between the *i*th neighbor star and the alignment star, and *n* is the number of the observed stars in the star pattern.

According to the centroid coordinates of the observed stars in the star pattern, the plane included angle between the neighbor star and the alignment star can be achieved using Equation (7).

(7) $$\theta_{i}={\text{tan}}^{-1}(y_{i}^{\prime }/x_{i}^{\prime })$$

It should be noted that the plane included angle between the neighbor star and the alignment star ranges from −180° to +180°. The centroid coordinates of the main star is (0, 0) in *o*ʹ*x*ʹ*y*ʹ coordinates, and the plane included angle of the alignment star is 0°.

The one-dimensional vector pattern not only expresses the position information of the observed stars, but also describes intuitively the information of the plane included angles between the neighbor stars and the alignment star, which can fully express the space geometry information of the observed stars in FOV.

## 3. Generation of the Feature Vector and the Process of Star Identification

### 3.1. Generation of the Feature Vector

**Figure 3:**
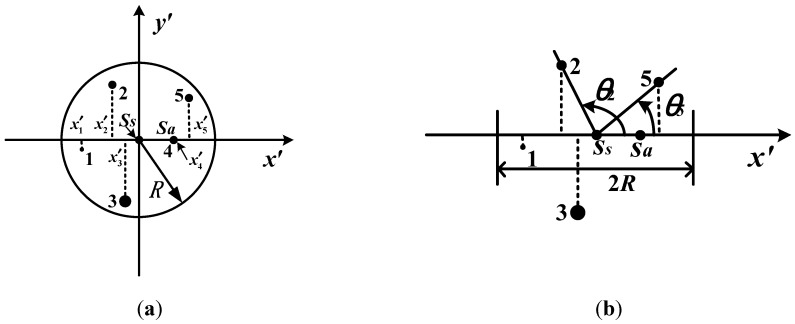
Generation of the one-dimensional vector pattern. (**a**) The positions of the observed stars on *o*ʹ*x*ʹ axis; (**b**) The plane included angles.

In order to enhance the robustness of the proposed algorithm, the *o*ʹ*x*ʹ axis within the scope of 2*R* is divided at regular intervals (see Figure 3b). Set the resolution of the *o*ʹ*x*ʹ axis locating in the neighboring region of the main star be *m*, so the interval on the *o*ʹ*x*ʹ axis will be 2*R*/*m*. The feature vector of the main star can be achieved according to the results of the one-dimensional vector pattern. So the feature vector of the main star can be expressed as

(8) $$pat(s)=\left(a_{1},a_{2}\ldots ,a_{m}\right)=\left\{a_{j}\right\},j=1,\ldots ,m$$

As described above, the pattern of the main star is expressed as a 1 × *m* vector *pat* (*s*). Each value in *pat* (*s*) indicates that whether there are observed stars whose horizontal axis coordinates are located in the corresponding interval or not. For every value in *pat* (*s*), that is

(9) $$a_{j}=\left\{ \begin{array}{cc} \theta_{i}, & x_{i}\in \left[(j-1)\times 2R/m,j\times 2R/m\right)\\ 0, & else \end{array}\right.,\;\;\;\;\;\left(x_{i},\theta_{i}\right)\in V,\;\;\;\;\;i\in \left(1,\ldots ,n\right),\;\;\;\;j\in \left(1,\ldots ,m\right)$$

where [(*j* − 1) × 2*R*/*m*, *j* × 2*R*/*m*) is the scope of the *j*th value in *pat* (*s*) on the *o*ʹ*x*ʹ axis, and *n* is the number of the observed stars in the star pattern, and *V* is the one-dimensional vector pattern of the star pattern.

If there is more than one observed star whose horizontal axis coordinates locate in the *j*th interval of the *o*ʹ*x*ʹ axis, the maximum plane included angle among these observed stars will be chosen as the value of *a_j_*. That is

(10) $$a_{j}=\text{max}\left\{\theta_{1},\ldots ,\theta_{p}\right\}$$

where *p* is the number of the observed stars whose horizontal axis coordinates locate in the *j*th interval of the *o*ʹ*x*ʹ axis. If there has no observed star whose horizontal axis coordinate locates in the *j*th interval, *a_j_* = 0.

### 3.2. Process of Star Identification

The feature vector of every navigation star can be achieved according to the extraction method of the feature vector described above, and all these feature vectors form the feature database of the navigation stars. During the process of star identification, one of the stars observed in FOV is chosen as the main star, which will be used to build the feature vector with its neighbor stars. Then the feature vector of the main star will be compared with the feature vectors in the feature database to find the matching star pattern.

Let the feature vector of the navigation star in feature database be *pat*(*c*), and the feature vector of the star observed in FOV be *pat*(*s*).The similarity between *pat*(*s*) and *pat*(*c*) is measured by the summation of the absolute differences of the two feature vectors. The summation of the absolute differences of the two feature vectors can be expressed as

(11) diff(pat(s),pat(c))=∑abs(pat(s)−pat(c)),      c=1,…,N

where *N* is the number of the navigation stars in the feature database. The smaller the summation, the more similar the two feature vectors become. As described above, the comparison of the two star patterns is converted into the comparison of the two vectors, which greatly simplifies the problem of star identification.

In order to accelerate star identification and avoid searching the entire feature database to find the matching star pattern, a search strategy is proposed to achieve the matching result as quickly as possible. That is the number of the non-zero values in the feature vector is used to constraint the search scope in the feature database.

Assume that the number of the non-zero values in *pat*(*s*) is *L_s_*, then the feature vectors in the feature database with the number of the non-zero values between *L_s_* − ε_1_ and *L_s_* + ε_2_ will be compared with *pat*(*s*). Therefore, it can quickly achieve the matching result with fewer comparisons instead of the searching through the entire feature database. So the process of star identification can be expressed as

(12) $$\begin{array}{c} result=\underset{c}{\text{min}}\left(diff\left(pat(s),\;\;\;pat(c)\right)\right),\;\;\;\;\;\;\;\;L_{c}\in [L_{s}-\varepsilon_{1},L_{s}+\varepsilon_{2}]\\ \underset{c}{\text{min}}\left(diff\left(pat(s),\;\;\;pat(c)\right)\right)\leq \varepsilon_{0} \end{array}$$

where *L_c_* is the number of the non-zero values in *pat*(c), ε_1_ and ε_2_ are the tolerances of the number of the non-zero values, and ε_0_ is the pre-set value. The difference between the best matching pattern and the main star pattern is smallest among the comparison results. Meanwhile, the smallest difference is not larger than the pre-set value.

From the above description, the record of every star pattern in the feature database can be expressed as

(13) $$m\text{Re}cord=\left\{id,\;\;\;L_{c},\;\;pat(c)\right\},\;\;\;\;\;\;c=1,\ldots ,N$$

where *id* is the index of the navigation star.

## 4. Simulation Conditions

### 4.1. Parameter Setting for Star Sensor

The Tycho-2 catalogue is used as the source of the navigation stars data. Some stars in the star catalogue may be lack of brightness or location information, so that they cannot be chosen as the navigation stars. Due to the limitation of the resolution of CCD in star sensor, it cannot clearly distinguish the two stars when they appear too close. In this paper, two stars apart less than 20 pixels apart (about 0.39°) are considered binary stars, which cannot be chosen as the navigation star. Therefore, there are 6685 stars to be chosen as the navigation stars whose apparent magnitudes range from 1.0 Mv to 6.5 Mv. Each of the navigation stars is chosen in turn as the optical axis direction of CCD to implement the process of star identification. The resolution of the simulated stellar image is 1024 × 1024 pixels with a 20° × 20° FOV. The average number of the stars observed in FOV is 30.32, with 68 as the maximum and 3 as the minimum. The experiments are carried out using a microcomputer with a 3.20 GHz Pentium (R) Dual-Core, 1.96 GB RAMS. All tests are carried out in the same simulation conditions.

The achievable performance of the proposed algorithm is demonstrated via a comparison study between the pyramid algorithm [8], the modified grid algorithm [20] and the LPT algorithm [28]. This paper has revealed the influence of the positional noise, the false stars, and the lost stars on the star identification in the four star identification algorithms. The complexity of the proposed algorithm was also compared with that of the other three algorithms.

Due to the characteristics of the star pattern, the observed stars close to the center of FOV are chosen as the main stars to complete the star identification. The attitude of the spacecraft can be calculated based on the position information of every three observed stars [1]. Therefore, the identification process is considered to be unsuccessful when the number of stars observed in FOV is less than three.

### 4.2. Selection of the Identification Parameters

As is well known, a lot of the geometric information about the observed stars located on the edge of FOV is lost. In order to obtain the geometry information out of the observed stars as much as possible, the observed stars near the center of FOV are chosen as the main stars in this paper. The resolution of the *o*ʹ*x*ʹ axis locating in the neighboring region of the main star is set to be *m* = 100. During the process of star identification, the tolerances of the number of the non-zero values are set to be ε_1_ = ε_2_ = 1, and the pre-set value ε_0_ is set to be 172 in experience.

The number of the observed stars in the neighbor region is different with the different neighborhood radius for the same main star. So the choice of the neighborhood radius *R* will affect the number of the neighbor stars of the main star, so as to affect the star pattern of the main star. Therefore, it is necessary to choose an appropriate neighborhood radius *R*. If *R* is too small, much geometric information of the stars observed in FOV will be ignored, and it cannot be possible to structure the unique pattern for every navigation star. If *R* is too large, the amount of the geometric information of the observed stars is too large to be easily manipulated. In addition to this, the star pattern is sensitive to noise when *R* is too large, which is rather obvious when the main star locates on the edge of the FOV.

**Figure 4:**
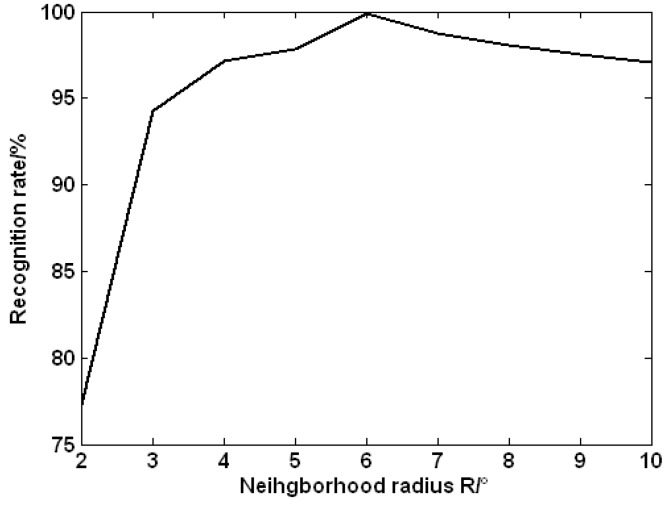
Recognition rate with different neighborhood radiuses *R*.

In this section, we discuss the recognition accuracy of the proposed algorithm under different neighborhood radiuses. Figure 4 shows the statistics results without any noise that every navigation star is adopted as the direction of the optical axis when *R* ranges from 3° to 10°. It can be found from Figure 4 that the recognition rate of the proposed algorithm changes with the increase of *R*. The recognition rate is very low when *R* is small, and subsequently the recognition rate increases with the increase of *R*. It achieves the largest recognition rate when *R* = 6°, then the recognition rate falls down with the increase of *R*. In the subsequent tests, the neighborhood radius is set to be *R* = 6°.

## 5. Experiment Results and Analysis

The evaluation criteria of the star identification algorithm mainly includes the influence of positional noise, false stars, lost stars on the recognition accuracy of star identification, the recognition speed of star identification, and memory usage. In this section, we describe the performance of the four star identification algorithms under the same simulation conditions.

### 5.1. Performance of Different Algorithms under Positional Noise

There are many different noises [39] in star sensors, and the positional noises are simplified in this paper. The positional noises are directly added into the actual centroid coordinates of the stars observed in FOV to investigate the robustness of the four star identification algorithms in terms of the positional noise.

Figure 4 shows the recognition rates of the four star identification algorithms in which only the positional noises are added. The results show that the anti-noise ability of the proposed algorithm is better than that of the pyramid algorithm, the modified grid algorithm, and the LPT algorithm. The recognition rate of the proposed algorithm is up to 98.55% when the positional noise is 0.5 pixels, while the recognition rates of the pyramid algorithm, the modified grid algorithm, and the LPT algorithm are just about 96.76%, 97.61%, and 91.76%, respectively.

**Figure 5:**
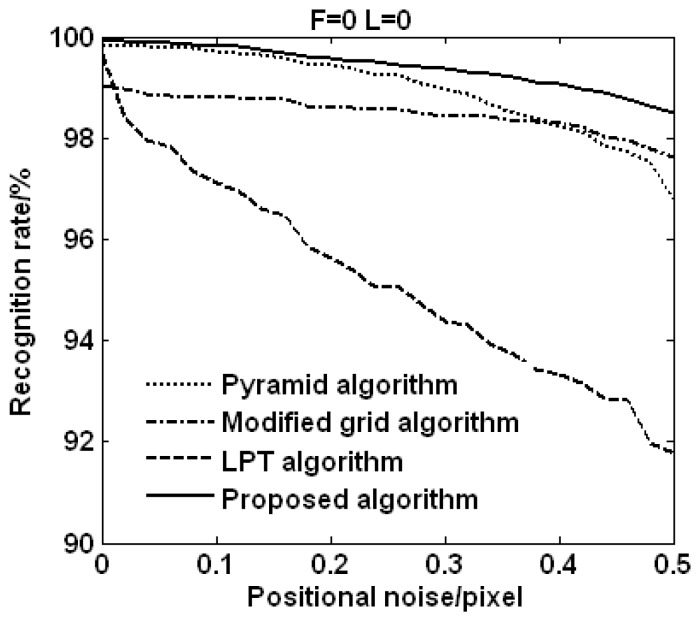
Recognition rates *vs.* positional noise (F: false star, L: lost star).

It can be known from Figure 5 that the recognition rate of the proposed algorithm is higher than that of the other three algorithms, and the downtrend of the recognition rate of the LPT algorithm is greater than that of the other three algorithms. With the increase of the positional noise, the recognition rate of the proposed algorithm decreases just about 1.37%, while the recognition rates of the pyramid algorithm, the modified grid algorithm, and the LPT algorithm decrease about 3.06%, 1.43%, and 7.89%, respectively. From the analysis data, it can be found that the downtrend of the modified grid algorithm is similar to that of the proposed algorithm with the increase of the positional noise, and the LPT algorithm is sensitive to the positional noise which has the biggest decline of 7.89%.

As can be seen by the above description, the proposed algorithm outperforms the pyramid algorithm, the modified grid algorithm, and the LPT algorithm in terms of the positional noise.

### 5.2. Performance of Different Algorithms under False Stars

Space debris and spacecraft, which may be mistaken as the observed stars (false stars), will affect the performance of the star identification algorithm. In this section, false stars will be added on the simulated stellar image to verify the performance of the star identification algorithm. The positions of false stars are random on the simulated stellar image.

**Figure 6:**
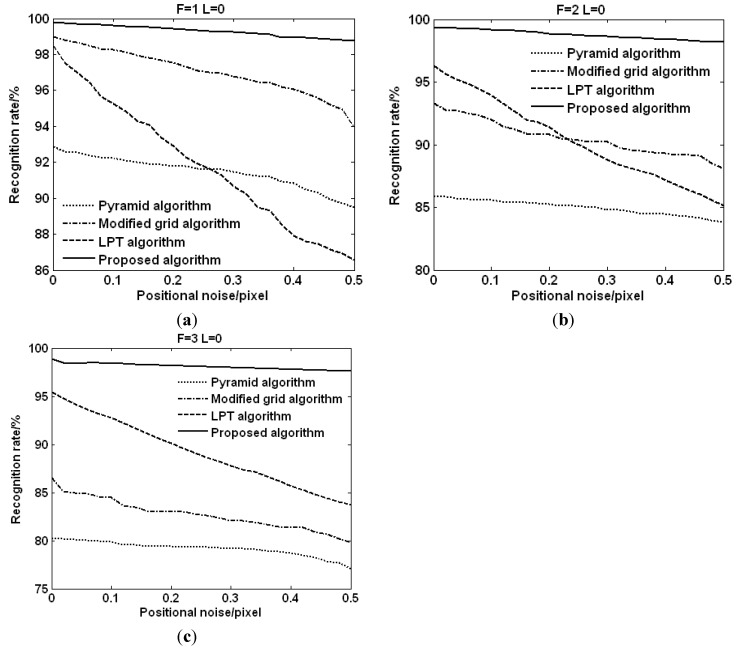
Recognition rate *vs.* false stars. (**a**) Recognition rate with 1 false star; (**b**) Recognition rate with 2 false stars; (**c**) Recognition rate with 3 false stars.

Figure 6 shows the statistical results of the recognition rates of the four algorithms for all 6685 navigation stars with the number of false stars ranging from 1 to 3. In Figure 6, without positional noise, the identification rate of the proposed algorithm decreases from 99.80% to 98.90% when the number of the false stars increases from 1 to 3. Under the same conditions, the rate of the pyramid algorithm decreases from 92.85% to 80.27%, and the rate of the modified grid algorithm decreases from 99% to 86.50%, and the rate of the LPT algorithm decreases from 98.47% to 95.46%. It can be found that the recognition rates of the four algorithms decrease 0.9%, 12.58%, 12.5%, and 3.01%, respectively.

As can be seen by the above analysis, the declines of the recognition rates of the pyramid algorithm and the modified grid algorithm are large with the increase of the number of the false stars, while the false stars have little impact on the recognition rate of the proposed algorithm. Therefore, the proposed algorithm outperforms the pyramid algorithm, the modified grid algorithm, and the LPT algorithm in terms of the false stars.

### 5.3. Performance of Different Algorithms under Lost Stars

Some stars in the sky may not be captured by CCD (lost stars) because of the obstruction of other spacecraft or the hardware failure of the star sensor. In tests, the observed stars on the simulated stellar image are deleted randomly. The observation is meaningless when excessive lost stars are deleted from the stellar image. So the number of the lost stars deleted from the stellar image ranges from 1 to 2 in this paper.

**Figure 7:**
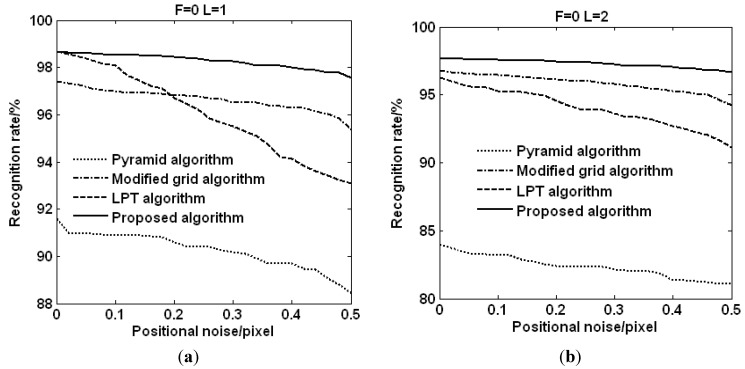
The recognition rates of different algorithm with false stars. (**a**) Recognition rate with 1 lost star; (**b**) Recognition rate with 2 lost stars.

Figure 7 shows the statistical results of the recognition rates of the four algorithms for all 6685 navigation stars with the number of the lost stars ranging from 1 to 2. From the results it can be found that the recognition rate of the proposed algorithm decreases from 98.67% to 97.7% when the lost stars are deleted without any positional noise. While the recognition rate of the pyramid algorithm decreases from 91.59% to 84%, and the modified grid algorithm decreases from 97.4% to 96.8%, and the LPT algorithm decreases from 98.67% to 96.27%. The declines of the four algorithms are 0.97%, 7.59%, 0.6% and 2.4%, respectively. Based on the analysis results, the pyramid algorithm is sensitive to the lost stars. Although the decline of the proposed algorithm is not as good as that of the modified grid algorithm, the recognition rate of the proposed algorithm is better than that of the other three algorithms.

As can be seen by the above description, the proposed algorithm outperforms the pyramid algorithm, the modified grid algorithm and the LPT algorithm in terms of the lost stars.

### 5.4. Identification Time and Memory Usage

The identification time and the memory usage should be taken into account in the practical application of the star identification algorithm. The identification times and the memory usages of the four star identification algorithms are summarized in Table 1.

**Table 1:** Identification time and memory usage of different algorithms.

Identification Algorithm	Max Time/s	Min Time/s	Average Time/s	Database Size
Pyramid	0.0610	4.0061 × 10^−^^4^	0.0275	130.57 KB
M. Grid	0.5886	0.0279	0.3946	7.38 MB
LPT	0.0811	0.0718	0.0738	665.89 KB
Proposed algorithm	0.0196	3.3496 × 10^−^^4^	0.0078	280.72 KB

The identification time of star identification is measured by the average identification time for the 3000 simulations. It can be found from the Table 1 that the proposed algorithm is most time-saving than the other three algorithms, which has an average identification time of 7.8 ms. In the proposed algorithm, the search scope of the matching pattern is constrained by the number of the non-zero values in the feature vector, which makes it able to achieve the identification result just with fewer comparisons. In addition, the feature vector of every star pattern is unique in the proposed algorithm, and the comparison of every two star pattern is simplified as the comparison of the two feature vectors.

In the modified grid algorithm, the comparison of two star patterns requires g × g logical AND operations, and the logical operations increase with the increase of the grid cells. Meanwhile, it needs to search the entire feature database to find the best matching pattern. Hence, the modified grid algorithm is time-consuming and its average identification time is longest at 0.3946 s.

The pyramid algorithm utilizes the *k-vector* approach to find the candidate matching results, which has no need to search the entire feature database to find the matching result, so it can accelerate the star identification to some extent.

The LPT algorithm has an average identification time of 73.8 ms, which is larger than that of the pyramid algorithm and smaller than that of the modified grid algorithm. The average identification time of the LPT algorithm is about 10 times of that of the proposed algorithm. In the Log-Polar algorithm, the feature vector needs to be shifted circularly for some time when two feature vectors are compared, which is time-consuming. Moreover, it needs to search the entire feature database to find the identification result.

In terms of the memory usage, the modified grid algorithm requires the most memory with a feature database of 7.38 MB, which will increase with the increase of the number of grid cells. The pyramid algorithm has the smallest feature database among these algorithms, because the dimension of its feature vector is lowest compared with the other algorithms. Although the feature database of the proposed algorithm is larger than that of the pyramid algorithm, it is acceptable and supported by the available hardware.

From the simulation results, we can make the conclusion that the proposed algorithm outperforms the pyramid algorithm, the modified grid algorithm, and the LPT algorithm in terms of positional noise, false stars, lost stars, and star recognition time. Despite the fact that the memory requirement for the proposed algorithm is more than that of the pyramid algorithm, it is supported by the available hardware.

## 6. Conclusions

An autonomous star identification algorithm has been proposed based on the one-dimensional vector pattern, in which the feature vector of the star pattern remains unchanged when the stellar image rotates. Compared with the pyramid algorithm, the modified grid algorithm, and the LPT algorithm, the star identification algorithm proposed in this paper has advantages of high recognition accuracy and good noise resistance ability. Moreover, the computational complexity of the proposed algorithm is lower than that of the other three star identification algorithms. The innovations of the proposed algorithm mainly include:

(1) Compared with the 0–1 string in the modified grid algorithm, the one-dimensional vector pattern can fully express the space geometry information of the stars observed in FOV, which makes a great contribution to star identification.
(2) The feature vector of the same observed star remains unchanged when the stellar image rotates, so the comparison of every two star pattern is simplified as the comparison of the two feature vectors, which can accelerate the speed of star identification.
(3) The utility of the number of the non-zero value in the feature vector can narrow down the search scope of the matching pattern, which can make it possible to achieve the matching result quickly in the feature database, instead of searching the entire feature database.
(4) Under the same conditions, the performance of the proposed algorithm is better than the other three star identification algorithms.
(5) Compared with the other three star identification algorithms, the proposed algorithm is simple and easy to replicate.

We have clearly validated the robustness and the effectiveness of the proposed algorithm by analyzing the experimental results and comparing the proposed algorithm with the other three star identification algorithms. Experiment results show that the proposed algorithm outperforms the pyramid algorithm, the modified grid algorithm, and the LPT algorithm in terms of identification time, recognition accuracy, and robustness. Moreover, the computational complexity of the proposed algorithm is lower than that of the other three algorithms. The proposed algorithm can promote the efficiency of the attitude determination for the spacecraft, which will expand the application of star sensors.
